# Need for Behavioral Interventions for Young Adults Living with Advanced Cancer in the U.S.

**DOI:** 10.3390/cancers16101910

**Published:** 2024-05-17

**Authors:** Lisa M. Gudenkauf, Rina S. Fox, Brian D. Gonzalez, Heather S. L. Jim, John M. Salsman, David E. Victorson, Stacy D. Sanford, Laura B. Oswald

**Affiliations:** 1 Department of Health Outcomes and Behavior, Moffitt Cancer Center, Tampa, FL 33216, USA; 2 Division of Advanced Nursing Practice and Science, University of Arizona College of Nursing, Tucson, AZ 85721, USA; 3 Cancer Prevention and Control Program, University of Arizona Cancer Center, Tucson, AZ 85719, USA; 4 Department of Social Sciences and Health Policy, Division of Public Health Sciences, Atrium Health Wake Forest Baptist Comprehensive Cancer Center, Wake Forest University School of Medicine, Winston-Salem, NC 27157, USA; 5 Department of Medical Social Sciences, Northwestern University Feinberg School of Medicine, Chicago, IL 60611, USA; 6 Cancer Control and Survivorship Program, Robert H. Lurie Comprehensive Cancer Center of Northwestern University, Chicago, IL 60611, USA

**Keywords:** young adult, advanced cancer, metastases, psychosocial support, behavioral intervention, behavior therapy

## Abstract

**Simple Summary:**

Young adults (YAs) aged 18–39 living with advanced cancer face unique and complex challenges, but tailored behavioral interventions remain scarce. This commentary identifies unmet psychosocial needs and offers recommendations for targeted interventions to help improve overall quality of care for the often-marginalized group of YAs living with advanced cancer.

**Abstract:**

The population of young adults (YAs) aged 18–39 living with advanced cancer is growing and faces a compounded set of challenges at the intersection of age and disease. Despite these substantial challenges, behavioral interventions tailored to YAs living with advanced cancer remain scarce. This commentary aims to (1) discuss the unmet psychological, social, and behavioral needs of YAs living with advanced cancer; (2) highlight the paucity of behavioral interventions tailored to this growing population; (3) offer recommendations for the development of behavioral interventions targeting the unique needs of YAs living with advanced cancer; and (4) describe potential far-reaching public health benefits of these targeted behavioral interventions.

## 1. Introduction

Behavioral interventions are a primary recommendation to address many psychosocial and physical consequences of cancer and cancer treatment among survivors of adult-onset cancer [1]. In contrast to typical medical interventions (e.g., medication, surgery, device), behavioral interventions can be broadly defined as interventions designed to improve physical and psychological health and well-being by influencing the actions an individual takes with regard to their own health, whether by intervening on actions specific to psychosocial well-being, healthy lifestyle behaviors, or other domains [2]. Behavioral interventions can be used to address multiple patient concerns simultaneously while also presenting a lower risk of side effects and the potential for less financial burden relative to other interventions targeting similar outcomes [1]. Evidence for the beneficial impact of behavioral interventions on psychological distress, healthy lifestyle behaviors, and quality of life, as well as on cancer outcomes via behavioral influences, has been well-established for decades [3]. Yet, the development and delivery of behavioral interventions has historically focused on adult cancer survivors and has only recently begun to address the specific needs of young adult (YA) survivors diagnosed between the ages of 18–39 years old [4].

Overall cancer incidence among YAs is on the rise in the United States, with more than 80,000 new cancer diagnoses and 9000 cancer-related deaths among YAs in 2020 alone [5,6]. Cancers are often diagnosed at later stages among YAs compared to other age groups for many reasons: cancer screening is not widely recommended in this age group, YAs might not receive regular medical care, and even when YAs present to a doctor, symptoms are often attributed to other causes [5,6]. After a cancer diagnosis, YAs can feel lost in the transition between pediatric and adult care settings and may encounter communication challenges with physicians who have less experience in treating YAs. YAs have higher uninsurance rates compared with the general adult population under age 65, and YAs may not be able to afford treatment or navigate supportive financial resources [5,7]. Partly due to these factors, cancer remains the leading disease-related cause of death among YAs [5]. Recognizing this, the National Comprehensive Cancer Network (NCCN) has published clinical practice guidelines for adolescent and YA oncology since 2012 [8], with the most recent update in 2024 [9].

Relative to screening-age adults, YAs are more likely to be diagnosed with advanced cancer [5], which is defined by the National Cancer Institute (NCI) as cancer that is unlikely to be cured but may be controlled for years with treatment. Overall, patients with advanced cancer are living longer with cancer as a chronic or terminal illness and may refer to themselves as “metavivors” [10,11]. Rates of advanced cancer at the time of diagnosis among YAs range from 1% for thyroid cancer to 43% for non-Hodgkin’s lymphoma [5]. However, these rates do not capture YAs that will later develop advanced disease due to disease progression or recurrence and, thus, are likely underestimates of the true prevalence of advanced cancer among YAs. Recognizing the challenges faced by individuals with advanced cancer, the NCI held a special meeting in 2021 to identify evidence gaps and opportunities to optimize survivorship care for this population [11].

Despite increased recognition of stresses experienced by the growing population of YA cancer survivors and the growing population of individuals living with advanced cancer, the specific population of YAs with advanced cancer have been largely overlooked in cancer survivorship research and underserved in clinical care. A critical gap is the lack of targeted behavioral interventions to address the unique challenges of YAs with cancer generally [12], and specifically for YAs with advanced cancer. This commentary aims to highlight the need for behavioral interventions for YAs living with advanced cancer in the United States by (1) discussing the unmet psychosocial and behavioral needs of this growing population, (2) highlighting the paucity of tailored behavioral interventions for YAs living with advanced cancer, (3) offering recommendations for the development of such behavioral interventions, and (4) describing the potential public health benefits of developing behavioral interventions for YAs living with advanced cancer.

## 2. YAs with Advanced Cancer Are a Unique Population with Distinct Psychosocial Needs

**Figure 1:**
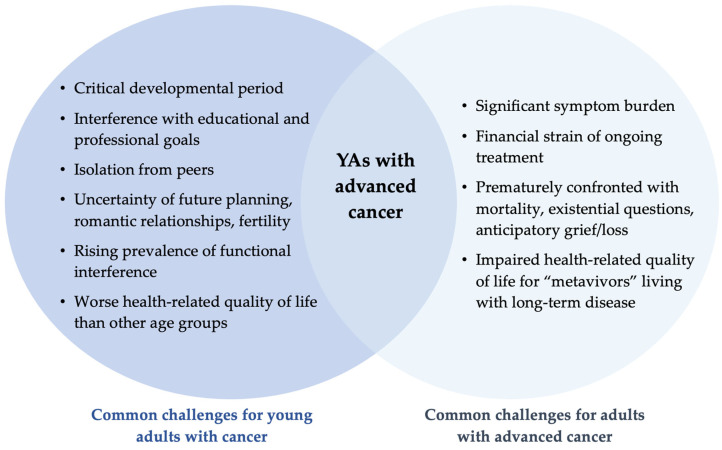
Compounded challenges of being a young adult (YA) with advanced cancer.

While cancer is distressing for individuals of any age and across all disease stages, YAs with advanced cancer confront a compounded set of challenges at the intersection of being a YA and having advanced disease (see Figure 1).

YAs with advanced cancer experience comparable life challenges and disruptions to those with non-metastatic cancer due to the diagnosis of cancer and treatment(s) during a critical developmental period [13,14,15,16]. YAs with cancer of any stage are often delayed in or derailed from achieving the socio-developmental milestones that typically characterize the transition from adolescence to adulthood, including achieving educational aspirations, launching professional careers, individuating from the nuclear family, and establishing romantic partnerships and families [17]. The excitement and hopefulness of young adulthood can be curtailed by cancer-related changes related to future planning, fertility, and relationship strain or social isolation [18]. YAs with cancer may struggle to connect with their peers without cancer as they simultaneously cope with side effects, uncertainty about the future, and impaired health-related quality of life (HRQOL). Indeed, the adjusted prevalence of functional limitations (e.g., difficulty with mobility or self-care) among YAs affected by cancer has risen over the past two decades, exceeding 55% by 2018, due in part to the addition of new therapies (e.g., targeted therapy, immunotherapy) to standard treatment regimens [19]. Due to the confluence of cancer-related and developmental challenges, YA cancer survivors tend to report worse HRQOL compared to other age groups [14,20].

In addition, similar to older adult populations living with advanced cancer, YAs with advanced cancer may experience significant symptom burden and financial strain due to ongoing cancer treatments that may not have a definitive end date [21,22]. They are prematurely confronted with their own mortality, among other untimely existential questions, and they may experience anticipatory grief and loss. For “metavivors” living long-term with advanced cancer as a chronic disease, the complex interplay of relevant biopsychosocial, behavioral, and clinical factors can substantially hinder HRQOL [10]. However, as it currently stands, these challenges remain poorly understood among YAs with advanced cancer, as very few studies have examined the lived experiences of this population.

YAs with cancer have previously been described as a “lost tribe” of patients with unmet needs [23,24], and, in response, many cancer centers across the US are developing YA-specific oncology programs. However, YA oncology programs have primarily focused on survivorship concerns among YAs receiving treatment with curative intent. Now, YAs living with advanced cancer could be considered a new “lost tribe” in the context of programs that were not designed to meet their unique combination of age- and disease-specific needs. For example, palliative care programs not initially designed for YAs are often ill-equipped to address complex and heterogenous psychosocial needs across the developmental span of YAs living with advanced cancer [25]. Given the vast developmental differences across the YA age continuum, YA care must recognize and address each patient’s individual level of maturity and independence [25]. There remains an obligation to address the significant and unique biopsychosocial challenges faced by YAs living with advanced cancer, and targeted efforts are needed to help bring this “lost tribe” into the fold of evidence-based psychosocial support services.

## 3. Behavioral Interventions Targeting YAs with Advanced Cancer Are Scarce

Despite the significant psychosocial needs of YAs with advanced cancer, there is a paucity of behavioral interventions tailored to this population, representing a concerning gap in the field of psycho-oncology. As it stands, the vast majority of behavioral interventions for cancer survivors have been developed for and tested among adults who have been diagnosed with cancer during adulthood, have completed active treatment with curative intent, and are currently living beyond cancer. For example, a review of systematic reviews evaluated the efficacy of behavioral interventions for improving HRQOL among adult cancer survivors [26]. It included 21 systematic reviews and more than 41,000 adult cancer survivors across 465 individual studies, of which 362 were randomized controlled trials (RCTs), underscoring the vast literature. Results showed that several behavioral interventions are efficacious for improving HRQOL in adult cancer survivors, including cognitive-behavioral therapy (CBT), mindfulness-based stress reduction (MBSR), and exercise-based interventions. Inclusion criteria among the systematic reviews required participants to be at least 18 years old. However, only 4 (19%) of the 21 systematic reviews reported a minimum age of participants in the YA age range, whereas the vast majority reported the minimum age of participants to be >40 years old. Moreover, it is not clear how many YAs were included in these systematic reviews, as only summary age ranges were provided.

Fewer studies have focused on behavioral interventions for individuals with advanced cancer, and most broadly included adult patients at least 18 years old. A 2019 systematic review evaluated the efficacy of behavioral interventions for adults (≥18 years old) with advanced cancer (i.e., ≥70% of study participants had stage 3 or 4 disease) published between 2007–2018 [27]. This systematic review included more than 7600 adults with advanced cancer across 68 RCTs evaluating various behavioral interventions (e.g., CBT, meaning-centered psychotherapy, dignity therapy). Overall, meaning-centered psychotherapy showed efficacy for improving HRQOL and helping patients with advanced cancer find meaning through enhanced communication. While promising, studies in this review were of variable methodological quality, and aspects of the included study designs limit the generalizability of findings. For example, some studies required participants to have a prognosis of at least 6 months, some required participants to have a highly functional performance status (e.g., ECOG performance status 0–2), and others limited recruitment to individuals aged ≥65 [27]. Moreover, the sample characteristics reported in this review did not specify the number of participants in the YA age category. Thus, the generalizability of findings to YAs with advanced cancer are unclear.

Even fewer studies have tested behavioral interventions targeting YAs with cancer. A 2017 systematic review of health promotion and behavioral interventions to improve health and well-being (e.g., quality of life, symptom burden, unmet needs) among YAs who had completed cancer treatment identified only 17 individual studies with 2314 cancer survivors aged 13–39 [28]. Of these 17 studies, only 10 RCTs were identified, which had variable quality and evident risk of bias. Small sample sizes and methodologic limitations precluded any conclusive evidence favoring specific intervention approaches, and the authors highlighted the dire need for high-quality, well-powered RCTs among individuals diagnosed as YAs, rather than survivors of childhood cancer. A subsequent 2020 integrative review again found only 17 articles directly pertaining to interventions to improve psychosocial outcomes (e.g., reduce distress, improve quality of life) among adolescent and YA cancer survivors during treatment [29]. Even within these scarce studies, the intervention approaches ranged broadly and generally promoted creative expression, peer interactions, individual coaching, and clinical interactions. Studies did not prominently feature evidence-based behavioral intervention approaches that have been well-established for improving psychosocial outcomes in older adults for decades (e.g., CBT, MBSR).

In contrast to the number of intervention studies for adults with cancer, individuals with advanced cancer, and the overall population of YAs with cancer, the current evidence-base of behavioral interventions for YAs with advanced cancer remains virtually non-existent. A 2023 systematic review of ClinicalTrials.gov revealed an increase in the number of behavioral intervention trials registered annually among adolescent and YA cancer survivors, from 2 per year between 2007–2014 to 11 per year between 2015–2021 [4]. Despite this promising trend, only 1 of the identified trials (i.e., <2% of YA-specific trials) targeted adolescents and YAs with advanced cancer [30]. Several additional areas for continued improvement were identified, such as the need for increased use of valid, standardized measures across the full range of HRQOL domains to assess intervention outcomes.

## 4. Considerations for Behavioral Intervention Development and Delivery

To move the field forward, it is essential to identify current challenges and potential solutions for developing and delivering tailored behavioral interventions for YAs with advanced cancer (see Table 1).

Indeed, several challenges and obstacles must be acknowledged and overcome to better engage YAs with advanced cancer in behavioral intervention research. This population is small relative to older adult populations and can receive care across both pediatric and adult care settings; this presents obstacles for research engagement and continuity of clinical care (e.g., transferring care across settings and changing care teams) [6]. In addition, factors, such as a high symptom burden, frequent hospitalizations, and death can contribute to study attrition, presenting challenges for research recruitment and retention efforts. YAs with advanced cancer also represent a diverse group with differing developmental needs in addition to varying cancer types, prognoses, and treatment experiences [4,28,29]. This heterogeneity can make it difficult to design one-size-fits-all intervention approaches in research and clinical practice, and there remains a need to better understand the diverse lived experiences of YAs with advanced disease at various times throughout the cancer continuum. In addition, resource allocation can present a challenge for YA program development [16], as healthcare systems may choose to prioritize age groups with a higher cancer prevalence or allocate limited resources to general psychosocial support programs. Such clinical resource allocation can result in fewer support services tailored to YAs overall and to YAs with advanced cancer specifically. Scientific team approaches are needed to overcome these research and clinical barriers to supporting YAs with advanced cancer as well as impacted loved ones and clinicians.

Some potential solutions already exist that can help address the common challenges and obstacles in effectively supporting YAs with advanced cancer. These solutions begin with cultivating greater understanding and awareness of this population’s unique psychosocial needs. Standardized use of biopsychosocial screening tools can help researchers and clinicians identify the most salient psychosocial needs of YAs with advanced cancer and properly allocate resources. Recruitment strategies can specifically target (rather than exclude) YAs and address the heterogeneity of this population by applying broad inclusion criteria. Partnerships with community sites, such as those available via the NCI Community Oncology Research Program (NCORP), can also help reach a more diverse and inclusive population of YAs. As a starting point, gold-standard evidence-based behavioral interventions can be adapted to the unique needs of and challenges faced by YAs with advanced cancer, such as holding space for focusing on dying well, in addition to living well with cancer. Tailoring evidence-based interventions would retain intervention fidelity while adapting delivery approaches and infusing content uniquely relevant to YAs [25]. Engaging key stakeholders (e.g., YAs with advanced cancer, care partners, clinicians) throughout the process of intervention and/or program development, as requested by YAs themselves [31,32], will be critical for producing interventions that reflect this population’s lived experiences, align with their preferences for intervention format and delivery, and can flexibly accommodate varying developmental needs, cancer types, and treatment experiences. From a resource allocation standpoint, implementation science approaches (e.g., hybrid effectiveness-implementation study designs) and cost-effectiveness analyses can help quantify the benefits of addressing unmet concerns in this high-need population who have the potential for elevated healthcare utilization across the lifespan.

## 5. Opportunities for Far-Reaching Public Health Impact

The benefits of developing behavioral interventions for YAs with advanced cancer are numerous. Behavioral interventions can be tailored to the unique needs, challenges, and circumstances of YAs with advanced cancer (e.g., relating content to life experiences specific to young adulthood, including vignettes and stories from other YAs, including images of YAs in patient-facing materials). Tailored behavioral interventions will help YAs with advanced cancer navigate complex emotional experiences, provide a safe space to express fears and concerns, contemplate ways to live out their values, and find meaning in the face of suffering. Interventions can offer a forum for peer support from other YAs facing similar challenges and foster a sense of belonging and understanding [11,33]. Evidence-based behavioral interventions (e.g., interventions grounded in CBT and acceptance and commitment therapy) could help YAs with advanced cancer develop and implement effective coping strategies to navigate uncertainty, manage treatment-related side effects, enhance HRQOL, and make informed choices about their care to balance medical treatments with personal goals and values [34,35]. Evidence-based approaches for addressing existential and end-of-life concerns (e.g., meaning-centered psychotherapy) could also be particularly beneficial for YAs with advanced cancer [27]. Finally, behavioral interventions can provide communication skills training to help address changes in personal relationships and effectively communicate their needs and preferences with their medical team to promote a holistic support system and patient-centered care [27,36]. Along with the development of tailored interventions, rigorous implementation science approaches can help illuminate unique dissemination considerations to streamline intervention delivery for YAs living with advanced cancer.

Beyond benefits to patients, tailored behavioral interventions for YAs with advanced cancer could indirectly benefit caregivers, clinicians, and the broader population. By providing an additional source of support and training in patient communication and interpersonal relationship skills, we can enhance patient–caregiver relationships and reduce stress and strain for caregivers [37,38]. Improved patient–provider communication and patient satisfaction can also help healthcare providers better meet the holistic needs of their patients with the potential for enhanced quality of care and patient outcomes [36]. Patient–provider interactions could be further improved by engaging clinicians as stakeholders in YA research, enhancing our understanding of the unique perspectives and lived experiences of clinicians caring for YAs with advanced disease. Finally, by addressing the unique needs of YAs with advanced cancer, interventions can contribute to enhancing public understanding and raising awareness of the struggles faced by this vulnerable population. This could help influence policy changes, increase research funding, and bolster support services both for YAs with advanced disease and for YAs with cancer in general [4]. Overall, by providing tailored behavioral intervention support, healthcare systems and society can demonstrate a commitment to addressing the unique challenges faced by YAs with advanced cancer. As clinical researchers, it is our duty to increase awareness, advocate for research funding, and encourage multidisciplinary care that effectively addresses the physical, emotional, and psychosocial aspects of receiving a life-limiting cancer diagnosis as a YA. Fulfilling this duty will help bridge the research-to-practice gap and improve overall quality of care for the often-marginalized group of YAs with advanced cancer.

## 6. Conclusions

This commentary highlights the dearth of behavioral interventions targeting the distinct concerns and needs of YAs with advanced cancer. We offer possible solutions to begin to address the challenges and obstacles in research and clinical care specifically tailored to YAs with advanced cancer. By optimizing intervention development and delivery, we can improve psychosocial outcomes, quality of life, and overall health for patients, caregivers, and clinicians, providing opportunities for far-reaching public health benefits in the U.S.

## Figures and Tables

**Table 1:** Summary of research and clinical challenges/obstacles and potential solutions for developing and delivering tailored behavioral interventions for young adults (YAs) with advanced cancer.

	Challenges/Obstacles	Potential Solutions
Research	The population of YAs is smaller than older adult populations. YAs are treated in both pediatric and adult care settings. Study attrition may be caused by high symptom burden, frequent hospitalizations, and death.	Target YAs with advanced cancer in recruitment efforts. Use broad inclusion criteria. Increase inclusivity and diversity through community partnerships. Continuously engage key stakeholders to assess needs.
Clinical	YAs are a heterogenous group with varying developmental needs, cancer types, prognoses, treatment experiences, and time in the cancer care continuum. Healthcare systems might prioritize age groups with a higher cancer prevalence or general psychosocial support programs.	Use standardized biopsychosocial screening tools to identify needs and allocate resources. Adapt evidence-based interventions. Use implementation science and cost-effectiveness analyses to quantify benefits.

## Data Availability

There are no original data directly related to the contents of this commentary.
